# Selenium nanoparticle rapidly synthesized by a novel highly selenite-tolerant strain *Proteus penneri* LAB-1

**DOI:** 10.1016/j.isci.2022.104904

**Published:** 2022-08-13

**Authors:** Mingshi Wang, Daihua Jiang, Xuejiao Huang

**Affiliations:** 1 Key Laboratory of (Guang Xi) Agricultural Environment and Products Safety, College of Agronomy, Guangxi University, Nanning 530004, China

**Keywords:** Microbiology, Microbial metabolism

## Abstract

Microorganisms with high selenite-tolerant and efficient reduction ability of selenite have seldom been reported. In this study, a highly selenite-resistant strain (up to 500 mM), isolated from lateritic red soil, was identified as *Proteus penneri* LAB-1. Remarkably, isolate LAB-1 reduced nearly 2 mM of selenite within 18 h with the production of selenium nanoparticles (SeNPs) at the beginning of the exponential phase. Moreover, *in vitro* selenite reduction activities of strain LAB-1 were detected in the membrane protein fraction with or without NADPH/NADH as electron donors. Strain LAB-1 transported selenite to the membrane via nitrate transport protein. The selenite was reduced to SeNPs through the glutathione pathway and the catalysis of nitrate reductase, and the glutathione pathway played the decisive role. *P. penneri* LAB-1 could be a potential candidate for the selenite bioremediation and SeNPs synthesis.

## Highlights

• A novel highly selenite-tolerant (up to 500mM) strain *Proteus penneri* LAB-1 was isolated
• More than 93% of 2mM SeO_3_^2−^ was reduced to Se^0^ by LAB-1 in 18 h
• LAB-1 transports SeO_3_^2−^ to its membrane by the nitrate transport protein
• SeO_3_^2−^ reduction takes place via glutathione pathway and catalysis of NR

## Graphical abstract

**Figure:**
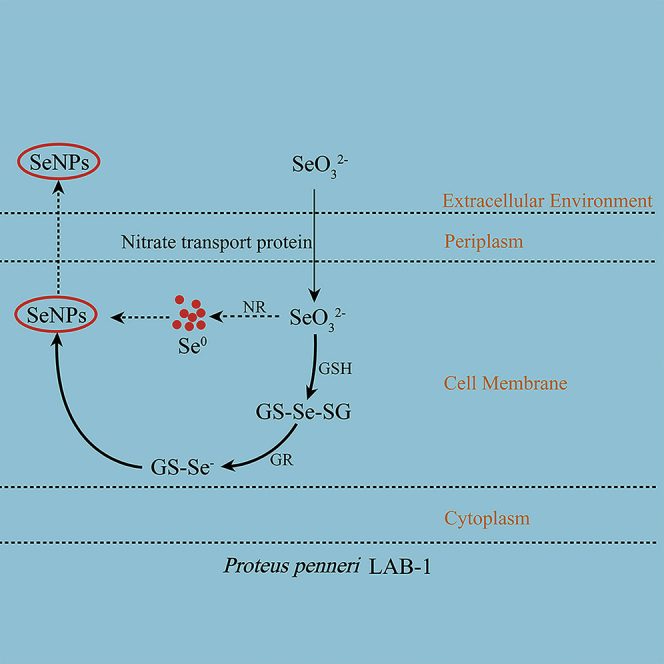

## Introduction

Selenium, a metalloid element, is an essential micronutrient for all living organisms (Lampis et al., 2017). However, it can be toxic at doses exceeding the recommended dietary limits (Fischer et al., 2020). Recent industrial developments have resulted in a high concentration of Se being released into the environment leading to pollution (Khamkhash et al., 2017; Khoei et al., 2017). The content and speciation of Se determine its toxicity (Huang et al., 2021). Usually, the valence state of Se in the environment varies, including II (selenide), 0 (elemental selenium and organic selenium species), IV (selenite), and VI (selenate) (Wang et al., 2018a). Among them, the water-soluble oxyanions selenate (SeO_4_^2−^) and selenite (SeO_3_^2−^) exhibit highly toxic effects on aquatic life (selenite is generally more toxic than selenate), whereas insoluble elemental selenium (Se^0^) exerts little or no toxicity (Avendano et al., 2016). Therefore, reduction of SeO_4_^2−^ or SeO_3_^2−^ to Se^0^ is an ideal strategy for the treatment of water or soil polluted with SeO_4_^2−^ or SeO_3_^2−^ (Rovira et al., 2008; Nancharaiah and Lens, 2015b; Wang et al., 2018b).

Various chemical, physical, and biological methods have been utilized to eliminate Se oxyanions (Rovira et al., 2008; Lu et al., 2018; Huang et al., 2021). Biological methods are generally preferred because of their eco-friendly characteristics, low cost, and ability to use self-generating catalysts (Wang et al., 2018b). A variety of microorganisms, such as *Pseudomonas putida* (Avendano et al., 2016), *Stenotrophomonas maltophilia* (Lampis et al., 2017), *Enterobacter cloacae* (Song et al., 2017), *Stenotrophomonas bentonitica* (Fresneda et al., 2018), *Alcaligenes faecalis* (Wang et al., 2018b), *Bacillus safensis* (Fischer et al., 2020), and *Providencia rettgeri* (Huang et al., 2021), reduce SeO_4_^2−^ or SeO_3_^2−^ to Se^0^. However, most of the reported Se-reducing bacteria exhibited relatively low SeO_4_^2−^ or SeO_3_^2−^ tolerance (<100 mM) and reduction efficiency (Tan et al., 2016; Fernandez-Llamosas et al., 2017; Zhu et al., 2018), although some isolates have exhibited extreme tolerance to SeO_3_^2−^ (>120 mM) and high SeO_3_^2−^ reduction efficiency (Saudi et al., 2009; Huang et al., 2021). Thus, isolating novel strains with high SeO_3_^2−^ tolerance and robust ability to reduce SeO_3_^2−^ is of the utmost importance. Furthermore, Se nanoparticles (SeNPs) biosynthesized by these microbes are gaining attention in the pharmaceutical, electronics, optics, and biomedical industries owing to their unique properties (Song et al., 2017).

SeO_3_^2−^ reduction and BioSeNP production could occur extracellularly, intracellularly, or both (Jain et al., 2016; Wang et al., 2018a; Fischer et al., 2020). Some bacteria reduce SeO_3_^2−^ to Se^0^ through enzymatic or non-enzymatic catalysis. Nitrate reductase (Nancharaiah and Lens, 2015a), thioredoxin reductase (Hunter, 2014), flavoprotein CsrF (Xia et al., 2018), and fumarate reductase (Song et al., 2017) catalyze this reduction. Biogenic glutathione, iron siderophores, and sulfides mainly catalyze the non-enzymatic pathway (Nancharaiah and Lens, 2015b). The SeO_3_^2−^ bioreduction mechanisms of various microorganisms are complicated and need to be thoroughly explored.

In this study, *P. penneri* LAB-1, which exhibits extreme tolerance to SeO_3_^2−^ (up to 500 mM), was isolated from lateritic red soil in Guangxi, China. A series of experiments was conducted to: (1) evaluate the ability of SeO_3_^2−^ reduction and SeNP production by strain LAB-1, (2) determine the site of SeO_3_^2−^ reduction, (3) clarify the pathway of SeO_3_^2−^ reduction, and (4) characterize SeNPs.

## Results and discussion

### Characterization and identification of strain LAB-1

**Figure 1:**
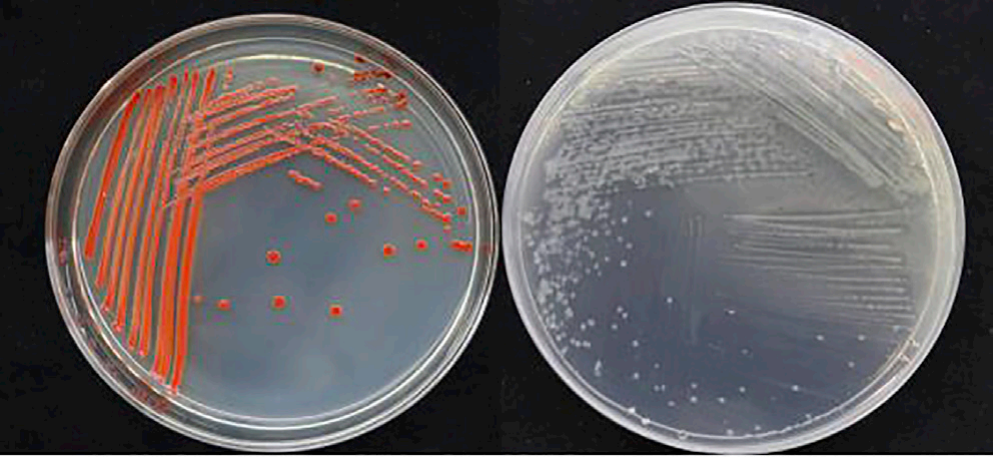
Images of cultures of strain LAB-1 grown in presence (left) and absence (right) of 2 mM selenite The red colony color indicates selenite reduction and the formation of elemental selenium.

**Figure 2:**
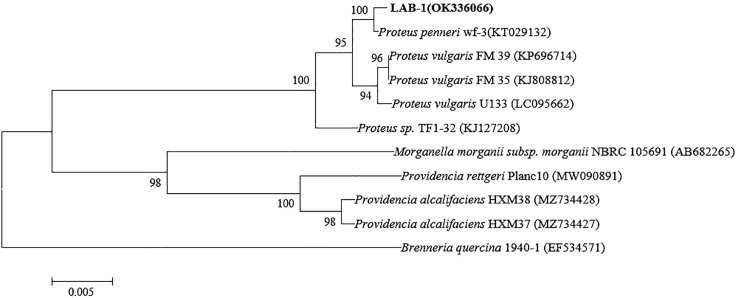
Maximum likelihood tree based on the 16S rRNA gene sequence of isolate LAB-1 The scale bars indicate 0.005 substitutions per site.

**Figure 3:**
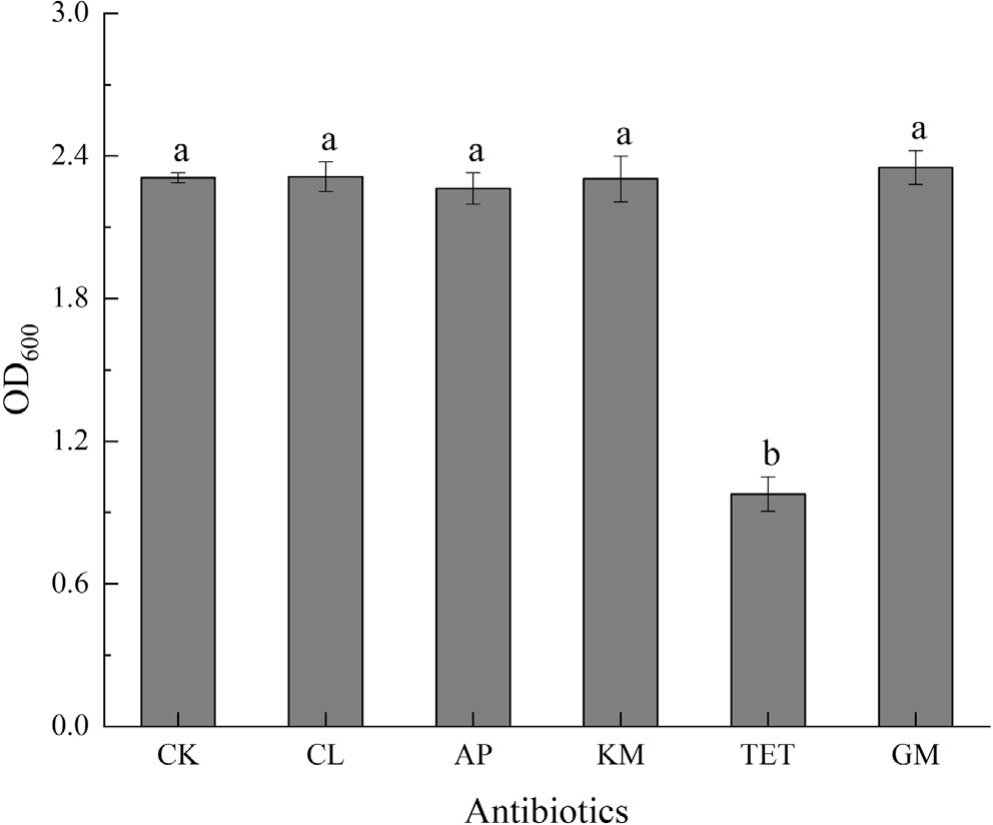
Influence of antibiotics (1 μg/mL) on *P. penneri* LAB-1 growth in 24 h (CK: no antibiotics; CL: chloramphenicol; AP: ampicillin; KM: kanamycin; TET: tetracycline; GM: gentamycin) Data are represented as mean, and different letters indicate significant differences between treatments at p < 0.05.

In this study, strain LAB-1, showing excellent ability of SeO_3_^2−^ tolerance and reduction, was isolated from Se-rich soil (Figure 1). LAB-1 grew even at a SeO_3_^2−^ concentration of 500 mM (Figure S1). Phylogenetic analysis showed that this strain was most closely related to the *P. penneri* wf-3 (KT029132) (Figure 2). Therefore, isolate LAB-1 was identified as *P. penneri* LAB-1 (OK336066). Moreover, most antibiotics (1 μg/mL), except tetracycline, did not inhibit the growth of isolate LAB-1 (Figure 3). This reveals that strain LAB-1 is resistant to many antibiotics. *Proteus* sp. is a rod-shaped Gram-negative bacterium commonly found in the environment (Drzewiecka, 2016). Some strains of *Proteus* sp. possess heavy metal resistance. For example, *Proteus* sp. H24 tolerates 1500 mg/L of chromium (VI) and completely reduces 1000 mg/L chromium (VI) within 144 h (Ge et al., 2013). *Proteus mirabilis* ZK1 tolerates 1400 mg/kg Zn^2+^ (Islam et al., 2014). *Proteus vulgaris* KNP3, isolated from soil, possesses a significant tolerance to Cu (1318 μM) (Rani et al., 2008). *P. mirabilis* YC801 reported by (Wang et al., 2018a) tolerates 100 mM SeO_3_^2−^. In the present study, we found that *P. penneri* LAB-1 showed a high SeO_3_^2−^ tolerance capacity (500 mM) and a high ability to reduce SeO_3_^2−^ to Se^0^ and synthesize SeNPs.

### Selenite reduction characteristics

#### Selenite reduction ability of strain LAB-1

**Figure 4:**
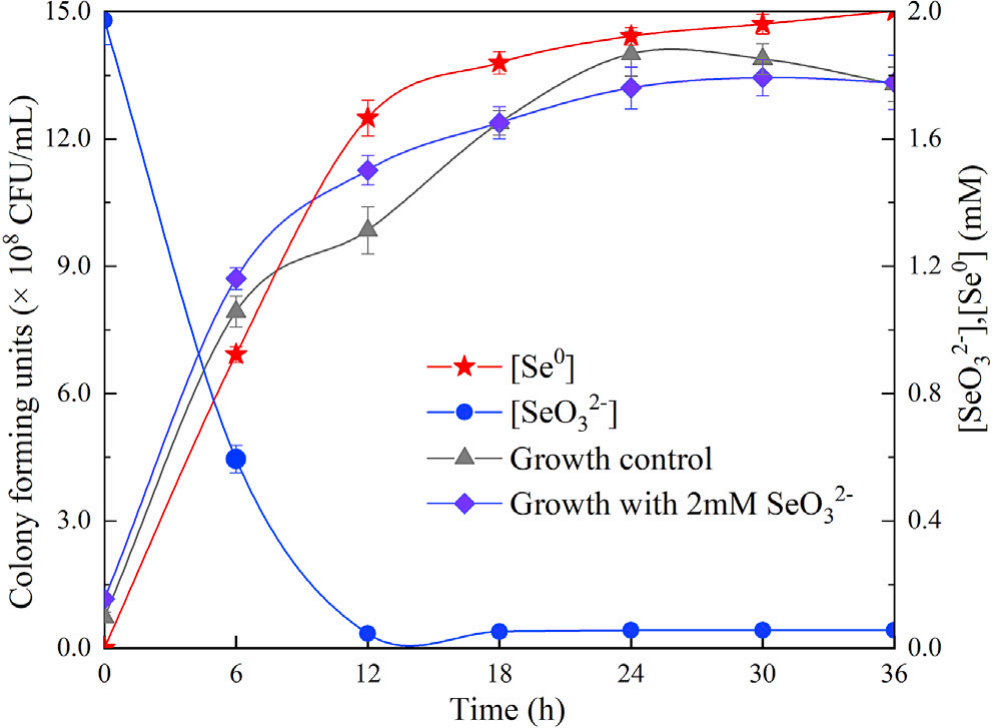
Time courses of bacterial growth, SeO_3_^2−^ removal, and Se^0^ formation by the strain *P. penneri* LAB-1 grown in LB medium containing 2 mM SeO_3_^2−^ Data are represented as mean.

As shown in Figure 4, the growth curves of strain LAB-1 in SeO_3_^2−^-containing medium showed the same pattern as that in the medium with no SeO_3_^2−^. This indicates that 2 mM SeO_3_^2−^ was not toxic to the isolate. However, the growth of *Bacillus mycoides* SeiTE01 (Lampis et al., 2014), *Streptomyces* sp. ES2-5 (Tan et al., 2016), and *S. maltophilia* SeITE02 (Lampis et al., 2017) was inhibited by 2 mM or even 1 mM SeO_3_^2−^. Thus, strain LAB-1 can be applied to SeO_3_^2−^-polluted water or soil bioremediation owing to its excellent SeO_3_^2−^ resistance. SeO_3_^2−^ decreased at the start of the growth phase (Figure 4), consistent with a report on *P. rettgeri* HF16-A growth (Huang et al., 2021). However, the SeO_3_^2−^ degradation performance of strain LAB-1 was contrast to *P. putida* KT2440 that converts SeO_3_^2−^ until it entered the mid-exponential phase (Avendano et al., 2016). Moreover, Se^0^ was produced once the SeO_3_^2−^ was reduced, and it could reach 46.75% and 93.27% after 6 and 18 h of cultivation (Figure 4). *P. mirabilis* YC801 (Wang et al., 2018a) and *S. maltophilia* SeITE02 (Lampis et al., 2017) required over 10 h (until the strains went into the exponential growth phase) to produce Se^0^ when cultured in 1 and 2 mM SeO_3_^2−^, respectively. These reveal that strain LAB-1 could rapidly reduce SeO_3_^2−^ to Se^0^.

#### Site of selenite reduction in strain LAB-1

**Figure 5:**
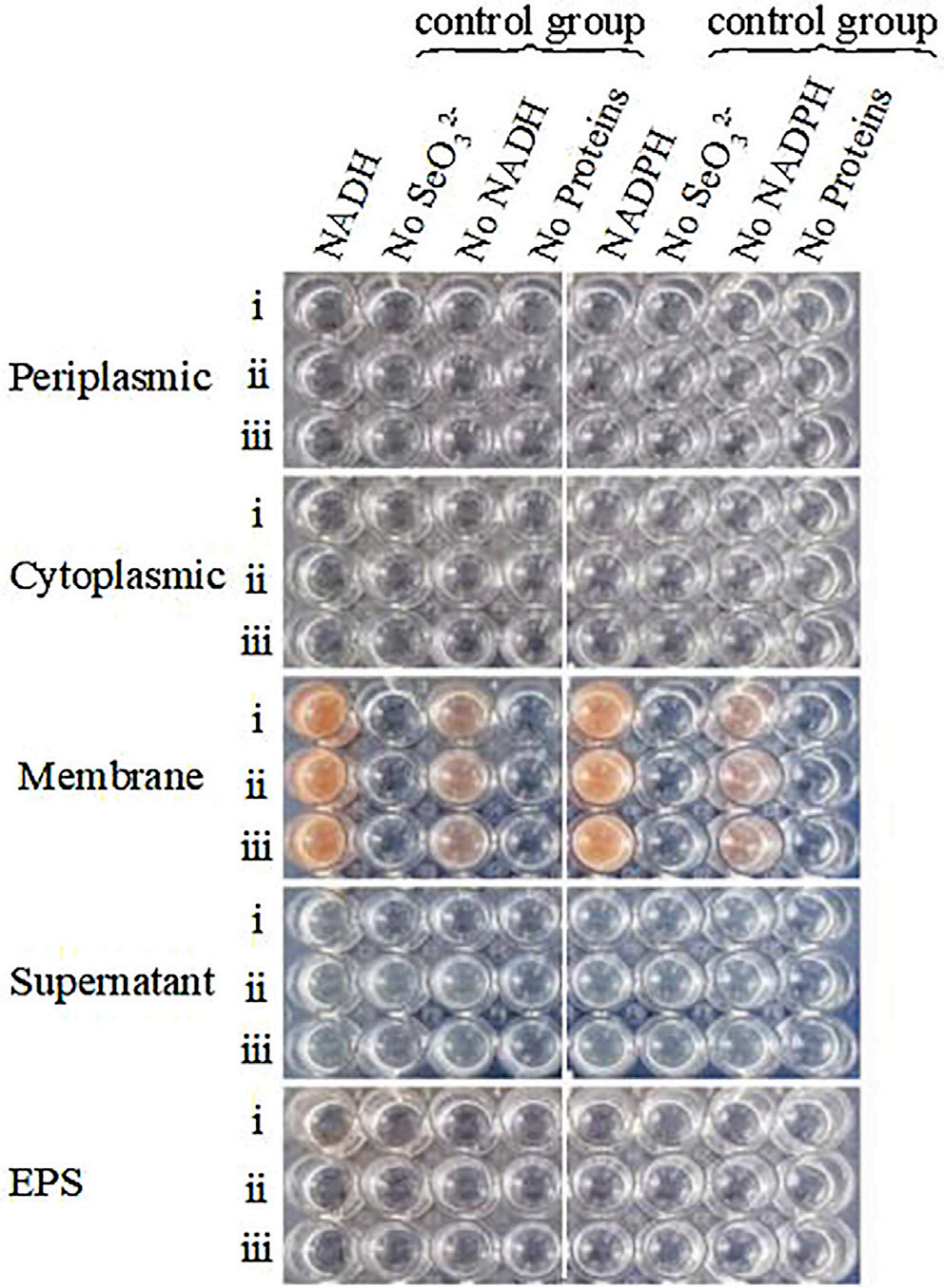
*In vitro* SeO_3_^2−^ reducing activity assays on different subcellular fractions (cytoplasmic, periplasmic, and membrane), supernatant, and exopolysaccharide (EPS) of *P. penneri* LAB-1 All experiments were performed in duplicate, with addition of 2 mM SeO_3_^2−^ and 2 mM NADPH or NADH. While 3 following control negatives were performed: without protein fractions, without selenite, without NADPH or NADH.

**Figure 6:**
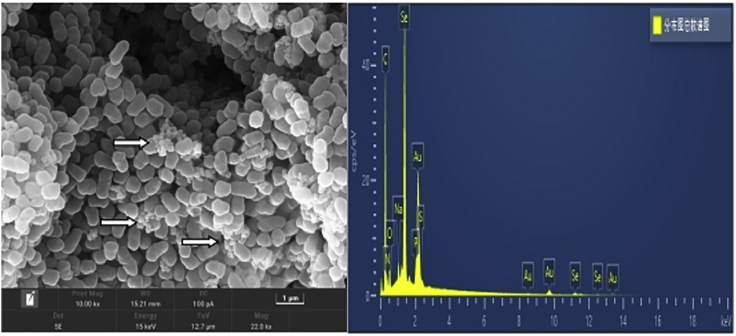
Scanning electron microscopy (SEM) analysis of *P. penneri* LAB-1 cultures grown in presence of 2 mM selenite (left) and the EDX analysis of purified SeNPs showing its selenium composition (Right)

**Figure 7:**
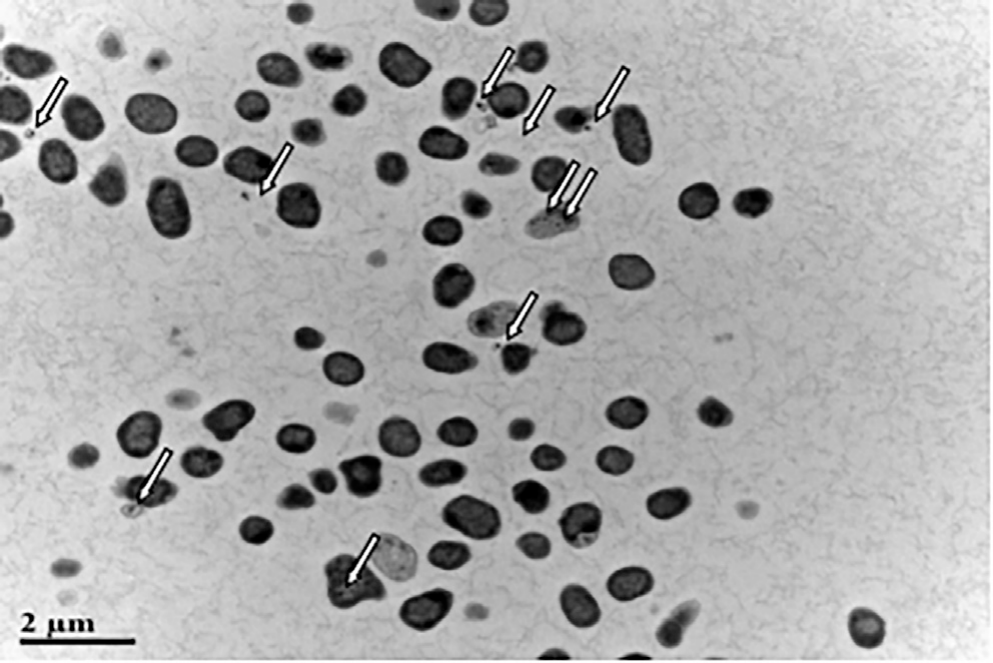
TEM micrographs showing SeNPs produced by *P. penneri* LAB-1 after 24 h of incubation with 2 mM sodium selenite

The SeO_3_^2−^ reduction activity of different fractions of strain LAB-1 is illustrated in Figure 5. It was clear that the intracellular SeO_3_^2−^ reduction in strain LAB-1 occurred in the membrane fraction. However, SeO_3_^2−^ reduction activity in *A. faecalis* Se03, *P. mirabilis* YC801, and *P. rettgeri* HF16 cells was localized in the cytoplasmic fraction (Wang et al., 2018a, 2018b; Huang et al., 2021). This indicated that the SeO_3_^2−^ reduction mechanism of strain LAB-1 was inconsistent with that reported by previous studies. The SeO_3_^2−^ reduction of LAB-1 occurred in the presence or absence of NADH or NADPH (Figure 5). Previous studies on *S. maltophilia* SeITE02 (Lampis et al., 2017) and *Burkholderia fungorum* strains (Khoei et al., 2017) revealed that SeO_3_^2−^ reduction activity only occurred with NADH or NADPH serving as an electron donor. We illustrated that the SeO_3_^2−^ reduction of *P. penneri* LAB-1 occurred in the membrane with or without NADH/NADPH. Moreover, SEM micrographs showed that SeNPs were found on the surface of *P. penneri* LAB-1 (Figure 6). TEM analysis clearly showed that the SeNPs were located in the extracellular spaces after 24 h of incubation (Figure 7). Thus, it is more likely that strain LAB-1 produces SeNPs within the cell and releases them into the medium.

#### Influence of additives and inhibitors on selenite reduction

**Figure 8:**
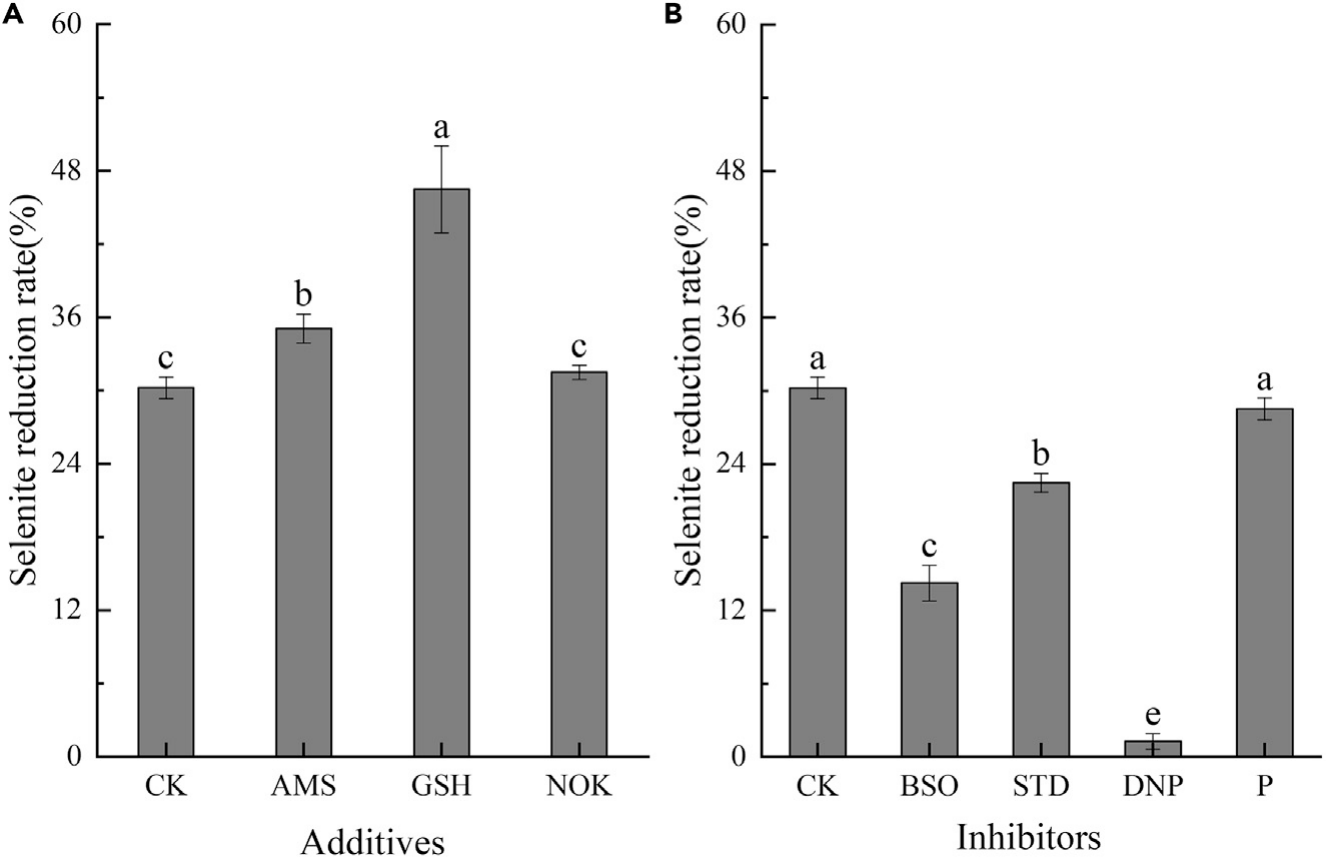
Impact of additive and inhibitor on selenite reduction by *P*. *penneri* LAB-1 Impact of additive (A) and inhibitor (B) on selenite reduction by *P. penneri* LAB-1 (CK: no additives or inhibitors adding; AMS: 2 mM potassium sulfate; GSH: 2 mM glutathione; NOK: 2 mM potassium nitrate; BSO: 6 mM L-butylthiocyanine-sulfoxide imine; STD: 30 mM sodium tungstate; DNP: 2 mM 2, 4-dinitrophenol; P: 2 mM Carboxypropylamine). Strain LAB-1 was incubated at 180 rpm and 30°C for 6 h. Data are represented as mean,and different letters indicate significant differences between treatments at p < 0.05.

Isolate LAB-1 reduces SeO_3_^2−^ to Se^0^ in the membrane (Figure 5). This illustrates that SeO_3_^2−^ in the medium was transported into the cell before being reduced. Further studies showed that SeO_3_^2−^ reduction was inhibited by the nitrate transport protein inhibitor 2, 4-dinitrophenol (Antonioli et al., 2007), although no change was observed with the sulfate transport inhibitor carboxypropylamine (Turner et al., 1998) (Figure 8B). This reveals that strain LAB-1 transports SeO_3_^2−^ into its cells through the nitrate transport protein.

The effects of additives and inhibitors on SeO_3_^2−^ reduction are displayed in Figure 8. The reduction rate of SeO_3_^2−^ increased by 16.24% (Figure 8A) when glutathione was added to the SeO_3_^2−^-containing medium but decreased by 16.00% when the glutathione inhibitor BSO was added (Figure 8B). This means that glutathione and glutathione reductase were involved in the reduction of SeO_3_^2−^ in the cells of strain LAB-1. The addition of BSO inhibited the production of glutathione, thereby reducing the reduction rate of SeO_3_^2−^. After adding glutathione, the glutathione content in the isolate increased, thereby promoting SeO_3_^2−^ reduction (Cui et al., 2016). However, the addition of BSO partially allowed the reduction of SeO_3_^2−^ by strain LAB-1, indicating the presence of other enzyme activities in the strain that catalyze the reduction of SeO_3_^2−^ to Se^0^. The reduction rate of SeO_3_^2−^ increased by 3.62% after adding potassium nitrate but decreased by 7.79% after adding sodium tungstate, a nitrate reductase inhibitor, indicating that nitrate reductase was also involved in the process. Therefore, strain LAB-1 reduced SeO_3_^2−^ to Se^0^ via the glutathione pathway, and this reaction was catalyzed by nitrate reductase.

#### Characterization of SeNPs produced by strain LAB-1

**Figure 9:**
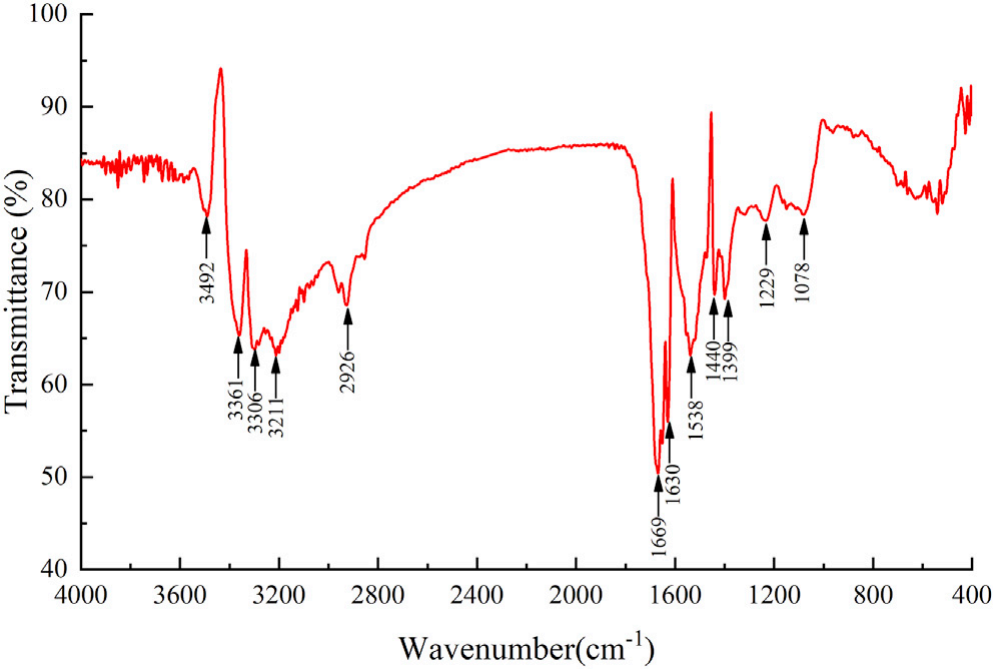
The FTIR spectrum of SeNPs synthesized by *P. penneri* LAB-1

Strain LAB-1 reduced SeO_3_^2−^ to Se^0^ and synthesized SeNPs (Figure 1). The produced SeNPs were spherical with an average hydrodynamic diameter of 274.9 ± 13.2 nm (Figure S2), covering the surface of the LAB-1 isolate (Figure 6). Energy-dispersive X-ray (EDX) analysis demonstrated the presence of Se, with Se-specific peaks observed at 1.39, 11.19, and 12.50 keV (Figure 6). Figure 9 shows the FTIR spectra of the SeNPs produced. The absorption bands at 3492, 3361, 3306, and 3211 cm^−1^ could be assigned to O‒ H/OH bonds or N‒H stretching and amide A of proteins, respectively (Xu et al., 2018). The peak centered at 1669 cm^−1^ was due to amide I, while that at 1538 cm^−1^ and 1630 cm^−1^ were attributed to amide II and amide III, respectively (Kamnev et al., 2017; Wang et al., 2018b). The peak at 1399 cm^−1^ was attributed to the stretching vibrations of COO^−^ (Lampis et al., 2017; Zonaro et al., 2017; Wang et al., 2018b). The peaks at 1229 cm^−1^ and 1078 cm^−1^ were assigned to the vibrations of C–O–C and C–O, respectively, indicating the presence of polysaccharides (Tugarova et al., 2018; Wang et al., 2018b). The results of FTIR analysis clearly showed that the surface of the SeNPs produced by *P. penneri* LAB-1 contained organic residues (carbohydrates, lipids, and proteins). Similar compositions of the organic groups on the appearance of SeNPs produced by *S. maltophilia* SeITE02 and *P. mirabilis* YC801 have already been reported (Lampis et al., 2017; Wang et al., 2018a). These organic groups participate in SeO_3_^2−^ reduction, SeNP formation, and the stabilization process.

### Conclusions

The novel, highly selenite-tolerant strain *P. penneri* LAB-1 was isolated from a naturally occurring Se-rich paddy soil. This strain transports selenite to its membrane via a nitrate transport protein. Then, selenite is reduced to Se^0^ via the glutathione pathway and catalyzed by nitrate reductase. The glutathione pathway plays the decisive role. More than 93% of 2 mM SeO_3_^2−^ was transformed to SeNPs and released into the extracellular space within 18 h. Our study is the first to report that *P. penneri* LAB-1 showed a high SeO_3_^2−^ tolerance capacity (500 mM) and the ability to reduce SeO_3_^2−^ to Se^0^ and synthesize SeNPs. Considering this high selenite resistance and robust capacity to synthesize SeNPs, LAB-1 will have potential applications in different biotechnological fields.

### Limitations of the study

In this study, a microorganism with high selenite resistance and effective selenite reduction ability was isolated. We just determined the main selenite reduction pathway of *P. penneri* LAB-1 through bacterial *in vitro* tests. It needs to continue in-depth analysis at the molecular level.

## STAR★Methods

### Key resources table

**Table:** 

REAGENT or RESOURCE	SOURCE	IDENTIFIER
**Chemicals, peptides, and recombinant proteins**		

Luria Bertani	Sigma	NA.85
Na_2_SeO_3_	Sigma	CAS:10102-18-8
Ethylenedinitrilotetraacetic acid	Sigma	CAS:60-00-4
Potassium nitrate	Sigma	CAS:7757-79-1
DNP	Sigma	CAS:51-28-5
Sodium tungstate dihydrate	Sigma	CAS:10213-10-2
Tetracycline	Solarbio	CAS:64-75-5
Ampicillin,	Solarbio	CAS:69-54-3
Chloramphenicol	Solarbio	CAS:56-75-5
Kanamycin	Solarbio	CAS:25389-94-0
Gentamycin	Solarbio	CAS:1405-41-0
Lysozyme	Solarbio	CAS:12650-88-3
DNase I	Solarbio	CAS:9003-98-9
NADH	Solarbio	CAS:606-68-8
NADPH	Solarbio	CAS:2646-71-1
Potassium in potassium suIfate	Solarbio	CAS:7778-80-5
L-Glutathione reduced	Solarbio	CAS:70-18-8
Probenecid	Solarbio	CAS:57-66-9
L-Buthionine-sulfoximine	Solarbio	CAS:83730-53-4

**Software and algorithms**		

Mega 7.0	Koichiro Tamura	https://www.megasoftware.net/
IBM SPSS Statistics 22	International Business Machines Crop	https://www.ibm.com/cn-zh/analytics

### Resource availability

#### Lead contact

Further information and requests for resources should be directed to and will be fulfilled by the lead contact, Xuejiao Huang (hxuejiao0412@sina.com).

#### Materials availability

This study did not generate new unique reagents.

### Experimental model and subject details

This study did not use experimental models typical in life sciences.

### Method details

#### Chemicals and culture medium

We used the Luria–Bertani (LB) medium (per liter, pH 7.0–7.2) for bacterial enrichment. This medium contains 10.00 g of NaCl, 10.00 g of tryptone, and 5.00 g of yeast extract. The Na_2_SeO_3_ solution was prepared in deionized water and sterilized through filtration.

#### Isolation and identification of selenite-reducing bacteria

The soil sample was obtained from a naturally occurring Se-rich paddy soil in Guangxi Province, southern China (23°06′34″ N, 107°43′30″ E). The total Se content of the soil was 0.58 mg/kg. One gram of the soil sample was suspended in 100 mL LB medium supplied with 1.00 mM SeO_3_^2−^ and incubated for 48 h (150 rpm, 30°C). The bacterial strains were subcultured thrice at an inoculum size of 5%. One hundred microliters of three dilutions (from 10^−5^ to 10^−7^) of the culture solution was inoculated onto LB plates containing 10.00 mM SeO_3_^2−^ and then cultured at 30°C for 24 h. Individual red colonies (indicating selenite reduction and Se^0^ formation) were streaked on new media to obtain pure isolates. Among the monocultures, isolate LAB-1 was used in this study owing to its sharp growth and excellent ability to reduce SeO_3_^2−^.

The cell morphology of strain LAB-1 was observed using an Olympus BH-2 optical microscope. We tested the antibiotic resistance of strain LAB-1 using tetracycline, ampicillin, chloramphenicol, kanamycin, and gentamycin. 16S rRNA gene fragment of the strain was amplified by PCR using 16S rRNA gene universal primers 27F (5′-AGAGTTTGATCCTGGCTCAG-3′) and 1492R(5′-TACGGCTACCTTGTACGACTT-3′). PCR reaction system (50 μL): primer 27F and primer 1492R (20 μmol/L) respectively 1.0 μL; DNA template 1.0 μL; mixed enzymes include dNTPs (2.5 mmol/L) 10.0 μL, 10 × Buffer 15.0 μL. Taq enzyme (5.0 U/μL) 1 μL, H_2_O 21 μL. The PCR reaction procedure is as follows: 96°C for 3 min; 93°C 30 s, 58°C 30 s, 72°C 60 s, 35 cycles; 72°C for 10 min. After the PCR reaction, 1% agarose was used for identification and Axygen gel recovery kit was used to recover the required PCR product fragments (Huang et al., 2018). The PCR amplification products of the strains were sequenced by general biological systems Co., Ltd. (Anhui). The sequence was compared with previously published bacterial 16S rRNA gene sequences in the NCBI database. Finally, the MEGA software (version 7.0) was used to construct a phylogenetic tree.

#### Selenite reduction characteristic of strain LAB-1

##### Sensitivity of selenite by strain LAB-1

Strain LAB-1 was activated in the LB medium and then inoculated into fresh LB medium containing 0–600 mM Na_2_SeO_3_ and cultured at 30°C and 150 rpm for 24 h. Then, 100 μL of culture cells was spotted onto LB agar plates and incubated for an additional 72 h at 30°C to clarify the content of SeO_3_^2−^ that inhibited the growth of isolate LAB-1 (Wang et al., 2018b).

##### Kinetic characteristics of selenite reduction by strain LAB-1

Strain LAB-1 was activated in the LB medium and inoculated into fresh LB medium containing 2 mM SeO_3_^2−^. The cultures without SeO_3_^2−^ and strain QZB-1 were used as controls. Cultures were cultivated at 30°C on a shaker (150 rpm) for 36 h. Bacterial growth was measured based on the number of viable cells (colony-forming units [CFUs]), and the concentrations of SeO_3_^2−^ and Se^0^ were measured. CFUs were determined by spreading 100 μL of the corresponding diluted samples on LB plates and incubating at 30°C for 72 h. The concentration of SeO_3_^2−^ was determined using an atomic fluorescence morphology analyzer (SA-20; Jitian, Beijing). The Se0 content was measured according to the spectrophotometric method (Khoei et al., 2017).

#### Localization of selenite reduction activity by isolates

To determine the location of SeO_3_^2−^ reduction in strain LAB-1 and to clarify the SeO_3_^2−^ reduction process, different fractions of isolate LAB-1 were collected, and activity assays were performed.

##### Intracellular fraction extraction

Isolate LAB-1 was cultured in the LB medium for 18 h and centrifuged at 10,000 × *g* for 10 min. The obtained pellets were treated with different reagents to extract the periplasmic and membrane fractions after being washed twice with 0.9% NaCl (Wang et al., 2018b).

##### Extracellular fraction extraction

For extracellular polymeric substance (EPS) extraction, isolate LAB-1 cultured in LB medium at 30°C for 5 d was collected and treated using the method described by Wang et al. (2018b). For supernatant preparation, isolated LAB-1 cultured for 18 h was centrifuged (10,000 × *g* for 10 min at 4°C). Following this, the supernatant was collected after being passed through a 0.22-μm filter (Khoei et al., 2017).

##### Selenite reduction activity test

The activity assays were performed in a 96-well plate. Each plate consisted of 100 μL bacterial fraction, 88 μL PBS, 10 μL SeO_3_^2−^ solution (2.0 mM), and 2 μL NADH or NADPH (electron donor, 2.0 mM). The plates were then incubated at 30°C for 72 h. Those lacking an electron donor, cell fraction (supernatant/cell protein/EPS), or SeO_3_^2−^ were used as negative controls (Khoei et al., 2017).

#### Using additives and inhibitors to explore the pathway of selenite reduction

Additives (potassium sulfate, glutathione, and potassium nitrate, 2 mM) were added to the LB medium with 2 mM SeO_3_^2−^. Media without additives served as controls. All experiments were conducted at 30°C and 150 rpm for 6 h. Then, samples were collected from the culture to determine the concentration of SeO_3_^2−^. Similarly, 2, 4-dinitrophenol (2 mM) and carboxypropylamine (2 mM) acted as transport inhibitors to explore the transport of selenite in the isolate LAB-1. L-butylthiocyanine-sulfoxide imine (BSO, 6 mM) and sodium tungstate (30 mM) were used as reductive inhibitors to study the selenite reduction pathway of isolate LAB-1.

#### Localization and characterization of SeNPs

Isolate LAB-1 was inoculated in LB medium and was either supplemented with 2 mM SeO_3_^2−^ or not and cultured at 30°C for 24 h. Then, the cultures were collected to prepare for transmission electron microscopy (TEM) according to the method described by Huang et al. (2021). The bacterial cultures were centrifuged (10,000 × *g* for 5 min at 4°C) to collect the pellets. Then, the pellets were resuspended in a pre-cooled fixative solution (2% glutaraldehyde in 0.1 M phosphate-buffered saline, pH 7.4) and fixed for 10 min at 4°C then centrifuged at 5000× *g* for 5 min. The pellets were embedded in the pre-cooled fixative solution again then fixed overnight at 4°C to do the TEM (JEM2000FXⅡ, Japan). To further characterize the SeNPs, scanning electron microscopy (SEM) and energy spectrum (EDS) analysis were performed as reported by Wang et al. (2018a). The pellets were collected according to the TEM analysis method above and fixed with 2.5% glutaraldehyde at 4°C for 24 h then dehydrated in ethanol solutions (30, 50, 70, 90%, and 100%) for 15 min per gradient step. Finally, the sample dried by an ultra-low temperature freezer (ALPHAL-4 LD PLUS) was examined with a Hitachi S4800 scanning electron microscope (Tokyo, Japan). To further characterize SeNPs, the LAB-1 cultures in 2 mM SeO_3_^2−^ medium were centrifuged at 10,000 × *g* for 10 min at 4°C. The resulting pellets were disrupted by ultrasonication for 10 min and then centrifuged at 40,000 × *g* for 40 min to harvest SeNPs. The hydrodynamic diameter of the SeNPs was analyzed using a Nano-ZS90X zeta potential particle size tester (Malvern, UK) (Lampis et al., 2017). The possible chemical bonds in the SeNPs were investigated using a Fourier transform infrared (FTIR) spectrophotometer after drying the purified SeNPs (Huang et al., 2018).

### Quantification and statistical analysis

We used SPSS version 22 to perform a one-way analysis of variance (ANOVA). Origin 8.6 was used to perform the graphical work. Different lowercase letters in the figure indicate significant differences under the same conditions (p < 0.05) (Figures 3 and 8).

## Data Availability

• The accession number for the genome assembly and raw reads reported in this paper is GenBank: OK336066. All data reported in this paper will be shared by the lead contact upon request. • This paper does not report original code. • Any additional information required to reanalyse the data reported in this paper is available from the lead contact upon request.
