# Supplementary material for: Selenium nanoparticle rapidly synthesized by a novel highly selenite-tolerant strain Proteus penneri LAB-1
Source: iScience. 2022 Aug 13;25(9):104904. doi: 10.1016/j.isci.2022.104904 (PMC9463581; doi:10.1016/j.isci.2022.104904)

iScience, Volume *25*

Supplemental information

Selenium nanoparticle rapidly synthesized by a novel highly selenite-tolerant strain *Proteus penneri* LAB-1

Mingshi Wang, Daihua Jiang, and Xuejiao Huang

SUPPLEMENTAL INFORMATION

Figure S1. Growth of strain LAB-1 incubated in different concentrations of selenite.

Related to Figure1.

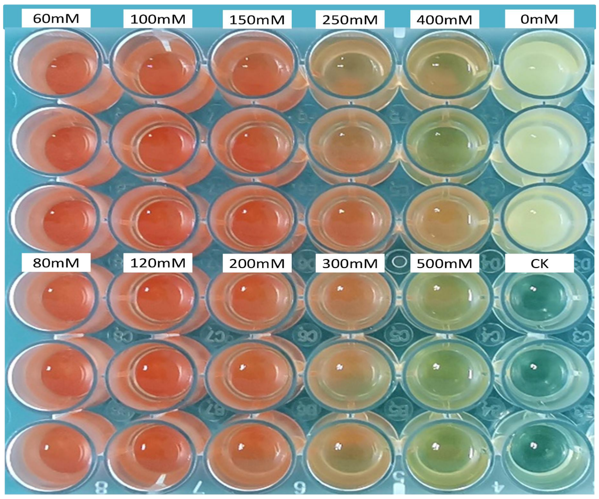

Figure S2. Size distribution of purified SeNPs.Related to Figure 6.

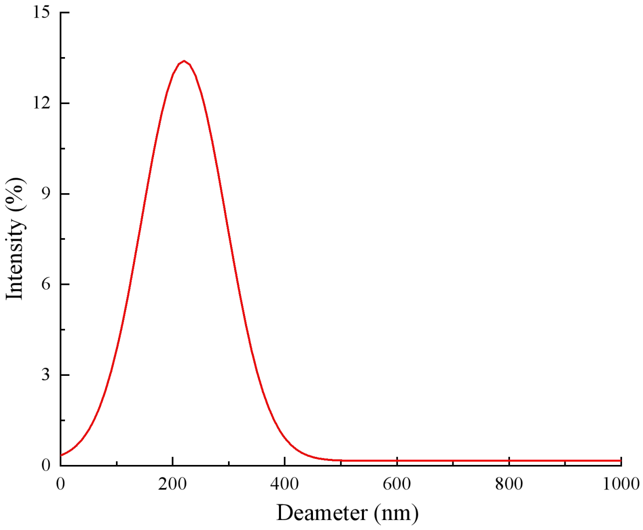

Supplement: Document S1. Figures S1 and S2 [file mmc1.pdf]
